# Nutritional Stunting Is Linked to Reduced Oral Microbiome Stability and Reconfigured Microbial Networks in Children: A Pilot Intervention Study

**DOI:** 10.3390/pathogens15060591

**Published:** 2026-05-31

**Authors:** Armelia Sari Widyarman, Nadeeka S. Udawatte, Swiluva Sigalovada Swilly Sumardy Ma, Citra Fragrantia Theodorea, Mario Richi, Wiwiek Poedjiastoeti, Chaminda Jayampath Seneviratne

**Affiliations:** 1 Department of Oral Biology, Faculty of Dentistry, Universitas Trisakti, Jakarta 11440, Indonesia; 2 School of Dentistry, The University of Queensland, Brisbane, QLD 4006, Australia; n.udawatte@uq.edu.au; 3 Tzu Chi School, Jakarta 14470, Indonesia; swiluva250@gmail.com; 4 Department of Oral Biology, Faculty of Dentistry, Universitas Indonesia, Depok 16424, Indonesia; citraob@gmail.com; 5 MiCORE Laboratory, Faculty of Dentistry, Universitas Trisakti, Jakarta 11440, Indonesia; mario.richi@trisakti.ac.id; 6 Department of Oral and Maxillofacial Surgery, Universitas Trisakti, Jakarta 11440, Indonesia; wiwiek@trisakti.ac.id

**Keywords:** childhood stunting, essential oil mouthwash, oral microbiome, probiotics, 16S rRNA sequencing

## Abstract

This non-randomized, open-labelled, controlled pilot trial investigated the impact of stunting on oral health and the oral microbiome, and evaluated the effect of 14-day probiotic or essential oil mouthwash interventions in children aged 8–12 years. Thirty-six participants (18 stunted, 18 non-stunted) were randomized into three parallel arms: probiotic lozenges (*Limosilactobacillus reuteri* DSM 17938 + ATCC PTA 5289), essential oil mouthwash, or water control. D-25OH level was assessed with ELISA, OHI-S, and PBI were examined, and oral microbiome was analyzed using 16S metagenomic sequencing. Stunted children demonstrated significantly higher gingival inflammation (PBI, *F* = 10.57, *p* = 0.002), reduced microbial alpha diversity, reductions in commensal *Streptococcus* spp., and increases in pathobionts, including *Parvimonas micra*, *Fusobacterium nucleatum*, and *Tannerella forsythia*. Beta-diversity analysis revealed distinct microbial communities (*p* = 0.001), with network analysis identifying these anaerobes as keystone hubs in stunted individuals. Salivary vitamin D and oral hygiene indices (OHI-S) also differed by stunting status. Fourteen-day interventions produced only modest, non-significant improvements in clinical indices and failed to induce significant shifts in microbial diversity or composition. These findings indicate that nutritional stunting is independently associated with oral dysbiosis and gingival inflammation. Short-term antiseptic interventions appear insufficient to reverse established microbial shifts, highlighting the need for sustained, integrated nutritional—oral health strategies.

## 1. Introduction

Stunting remains a major public health challenge in numerous socioeconomically challenged countries, affecting millions of children worldwide. Defined as the impaired linear growth resulting from chronic undernutrition, recurring infections, and persistent systemic inflammation during early life, stunting is now shown by emerging evidence to be associated with compromised oral health, in addition to its well-recognized consequences on physical and cognitive development [1]. Children affected by stunting frequently exhibit alterations in their salivary composition and flow rate, alongside disruptions in their oral microbiome. Such changes significantly increase their susceptibility to dental caries and periodontitis [2]. Chronic undernutrition is further associated with a sustained inflammatory burden, which can exacerbate periodontal tissue breakdown and impair the host immune response. This establishes a bidirectional relationship in which poor nutritional status predisposes children to oral infections, while chronic inflammatory conditions in the oral cavity may, in turn, aggravate systemic nutritional deficiencies [3]. Collectively, these observations support and emphasize the importance of evaluating oral health as an integral component of the broader pathophysiology of childhood stunting.

Oral hygiene and periodontal status are crucial indicators of oral health in children when their oral ecosystem and immune system are still developing. The Oral Hygiene Index-Simplified (OHI-S) is a widely used quantitative measure of dental plaque accumulation and calculus deposition, revealing the effectiveness of daily oral hygiene practices [4]. On the other hand, the Papillary Bleeding Index (PBI) provides a sensitive measure of gingival inflammation and early periodontal disease, as bleeding on probing can indicate the inflammatory status of the gingival tissues [4]. Together, the OHI-S and PBI are often used to track periodontal health and evaluate the response to clinical or behavioral interventions. In children with stunting, these indicators frequently exhibit poorer outcomes, possibly attributable to impaired immune function and altered oral microbiota composition, reinforcing the need for targeted oral health strategies in this vulnerable population [5].

Vitamin D deficiency has been strongly linked to both stunting and periodontal disease [6]. Vitamin D is an immunomodulatory hormone responsible for calcium homeostasis, regulation of antimicrobial peptide expression, and control of inflammatory response [6]. Insufficient vitamin D levels have been linked to increased periodontal breakdown, reduced salivary defense mechanisms, and unfavorable shifts in the oral microbiome [6]. Given that children with stunting frequently experience vitamin D deficiencies, concurrent assessment of vitamin D status alongside oral health markers and microbiome signatures may provide deeper insights into metabolic and microbiological interactions that influence disease susceptibility [7].

Recently, natural preventive strategies, such as probiotics and essential oils, have attracted growing interest for their potential to modulate the oral microbiome composition and improve periodontal outcomes [8]. Probiotics may promote the establishment of beneficial microbial communities capable of inhibiting pathogens and attenuating inflammation [8]. Similarly, essential oils possess antimicrobial, antioxidant, and anti-inflammatory properties that can disrupt biofilm formation and enhance oral hygiene [9]. The combined use of probiotics and essential oils has been proposed as a potentially synergistic approach for influencing oral microbial ecology while also affecting systemic biomarkers, including vitamin D.

Despite increasing interest in natural, microbiome-based interventions [10,11,12], there is limited evidence evaluating the effect of simultaneous probiotic and essential oils interventions on oral hygiene indices, periodontal inflammation, vitamin D levels, and oral microbiome composition, particularly in children with stunting, a population characterized by distinct biological and immunological vulnerabilities.

Therefore, this pilot intervention study aimed to evaluate changes in the OHI-S, the PBI, and oral microbiome profiles using next-generation sequencing (NGS), as well as salivary vitamin D levels (measured via ELISA), following a probiotic and essential oil intervention in both stunting and non-stunting children. This study seeks to provide novel insights into the interplay between nutritional status, oral microbiome status, and host factors, consequently contributing to the development of integrated oral-systemic health strategies for vulnerable pediatric populations.

## 2. Materials and Methods

### 2.1. Study Design

This study was conducted as a non-randomized, open-label, parallel-group controlled pilot trial with three independent arms. Participants were assigned to one of three arms: the intervention group receiving probiotic lozenges (BioGaia, Stockholm, Sweden; each lozenges contained *Limolactobacillus reuteri* DSM 17938 and *L. reuteri* ATCC PTA 5289), the intervention group receiving an essential oil mouthwash (Listerine Cool Mint, Kenvue, Summit, NJ, USA; active ingredients: thymol 0.064%, eucalyptol 0.092%, methyl salicylate 0.060%, menthol 0.042% *v*/*v* in 21.6% ethanol), and a negative control group receiving drinking water. Owing to the open-label design, both participants and investigators were aware of the intervention received. This parallel design was chosen to independently compare the efficacy of each intervention across outcome variables, avoiding the carry-over effects inherent in crossover designs. Subjects provided three biological samples, which were saliva, dental plaque, and gingival fluid (gum fluid), at baseline (the day before treatment) and 14 days post-intervention. The assigned interventions were administered by the subjects daily for two weeks after breakfast and regular teeth brushing. Sample collection took approximately 15 min and was conducted at the SDN 01 Cisarua, Kampung Ilmu, Purwakarta. Due to the strictly voluntary nature of participation in this study, subjects were allowed to withdraw at any time, with subject data confidentiality strictly maintained.

### 2.2. Sample

The study population consisted of children aged 8–12 years, with children classified as stunting (*n* = 36) and a healthy control group (*n* = 36). Before the study, informed consent was obtained from the parents or legal guardians of all participants. Exclusion criteria included children who had consumed antibiotics within 1–2 months before sampling, had been diagnosed with chronic systemic diseases, or had known allergies to probiotics.

### 2.3. Sampling

Three types of samples were collected from the subjects. To standardize oral hygiene practices, participants were provided with a uniform toothpaste and toothbrush throughout the study period.

#### 2.3.1. Saliva

Subjects were asked to sit with their heads tilted forward and their mouths opened, enabling saliva to drip passively from their lower lips. Unstimulated saliva was collected using a pre-weighed sterile tube. Participants were instructed to avoid brushing their teeth for at least 2 h before collection. Saliva was collected between 8:00 a.m. and 9:00 a.m., with a maximum total saliva volume of three (3) mL obtained from each participant.

#### 2.3.2. Gingival Fluid

Gingival fluid samples were collected using absorbent paper strips that were carefully placed into selected locations. The strips were removed after approximately 30 s, or once saturation was observed, which indicated fluid absorption.

#### 2.3.3. Dental Plaque

Plaque samples were obtained by carefully swabbing all surfaces of the mandibular molars using a sterile toothpick.

### 2.4. Measured Parameters

Salivary vitamin D levels were assayed using an ELISA according to the manufacturer’s instructions (Bioenzy, Jakarta, Indonesia). Briefly, the saliva samples were added to wells pre-coated with human 25-OH D antibody, followed by the addition of biotinylated human 25-OH D antibody to bind 25-OH D in the saliva. Streptavidin-HRP was added to bind the biotinylated antibody, and the plate was subsequently incubated at 37 °C for 1 h. Post-incubation, unbound streptavidin-HRP was washed away during five washing steps. A substrate solution was added after washing, and the color developed in proportion to the amount of human 25-OH D in the sample. The reaction was terminated by the addition of a stop solution, followed by absorbance measurement at 450 nm using a spectrophotometer (SAFAS MP96, Monaco).

Oral Hygiene Assessment (OHI-S) and Periodontal Assessment (PBI) were assessed by two trained and calibrated dentists. Inter-examiner reliability was established prior to data collection; the intra-class correlation coefficient (ICC) and Cohen’s Kappa (κ) were calculated for OHI-S and PBI scoring, with acceptable agreement defined as κ ≥ 0.80. This calibration procedure was conducted to minimize assessment bias and ensure accurate oral health comparisons between baseline and post-treatment assessments. Oral hygiene was assessed using the Simplified Oral Hygiene Index (OHI-S) according to Greene and Vermillion. The OHI-S comprises the Debris Index Simplified (DI-S) and Calculus Index Simplified (CI-S) and was recorded on six index teeth. For the DI-S, debris was evaluated by placing the probe at the incisal or occlusal third and moving it toward the cervical third of the tooth surface. Each tooth was scored from 0 to 3 based on the extent of soft debris coverage. The DI-S score was calculated as the sum of debris scores divided by the number of examined teeth.

For the CI-S, calculus was assessed by inserting the probe into the distal gingival sulcus and moving subgingival toward the mesial surface. Each tooth was scored from 0 to 3 according to the extent of supra- and subgingival calculus. The CI-S score was calculated as the sum of calculus scores divided by the number of examined teeth.

The OHI-S score was obtained by summing DI-S and CI-S values and was interpreted as follows:

0.0–1.2 = good oral hygiene;

1.3–3.0 = fair oral hygiene;

3.1–6.0 = poor oral hygiene.

Periodontal status was evaluated using the simplified Papilla Bleeding Index (PBI) for children aged 8–12 years in mixed dentition. A WHO 621 periodontal probe (0.5 mm ball tip; black band at 3.5–5.5 mm) was used. Six index teeth (16, 11, 26, 46, 31, 36) were examined. Probing was performed with approximately 20 g force using circumferential walking strokes at the buccal surfaces of 16, 11, 26, 31, and lingual surfaces of 36 and 46. Bleeding was recorded if present within 15 s after gentle probing.

Codes:

0 = no bleeding on probing (BOP), no calculus/retentive factors, probing depth < 3.5 mm;

1 = BOP present, no calculus/retentive factors, probing depth < 3.5 mm;

2 = supra-/subgingival calculus or biofilm-retentive factors, probing depth < 3.5 mm.

Microbiome profiling was performed using targeted next-generation sequencing of the 16S rRNA gene, as documented in the study by Widyarman et al. in 2022, with modifications [13]. In summary, DNA extraction from the plaque sample was conducted using Quick-DNA™ Miniprep Plus Kit (Zymo, Irvine, CA, USA) following the manufacturer’s instructions. The quality of DNA was checked using Qubit 3.0 Fluorometer (Invitrogen, Waltham, MA, USA). During library preparation, two PCR stages were performed: 16S rRNA PCR and Index PCR (UD Index Set A, Illumina, San Diego, CA, USA), each followed by a PCR Clean Up using Ampure XP Beads (Beckman Coulter, Indianapolis, IN, USA). Each PCR Clean Up had quality control check-ups with 1% agarose gel electrophoresis at 100 V for 30 min. After finishing both PCR stages, the amplified library was pooled and normalized to the required concentration of 80 pM as per the manufacturers’ instructions. An internal 20% spike-in control using phi-X v3 library (Illumina, San Diego, CA, USA) was combined and pooled together with the normalized library pool. Sequencing was performed using the iSeq 100 instrument using i1 v2 reagent (Illumina, San Diego, CA, USA) along with the parameter settings of 2 × 150 paired-end cycles and an additional 10 paired-end cycles for each index.

16S rRNA gene amplicon sequencing was performed to characterize bacterial community composition and its variation between stunted and non-stunted subjects following dietary interventions (probiotic and essential oil intake) at two time points (baseline and Day 14). Raw sequencing runs were initially assessed for analytical quality using Illumina Sequencing Analysis Viewer (v2.4.7) [14]. Paired-end reads were generated, and forward and reverse reads were merged to remove sequences containing ambiguous nucleotides (N). Quality filtering retained sequences longer than 200 bp, after which chimeric reads were identified and removed. High-quality sequences were subsequently clustered into operational taxonomic units (OTUs) using VSEARCH (v1.9.6) with a 97% sequence similarity threshold. Representative OTU sequences were taxonomically assigned by comparison against the SILVA 16S rRNA reference database, using the Ribosomal Database Project (RDP) Bayesian classifier. Community composition was then evaluated across multiple taxonomic ranks. In total, 862 OTUs were generated using an open-reference OTU picking approach, and rarefaction analyses indicated sufficient sequencing depth for downstream analyses.

Alpha diversity metrics, including Chao1, Shannon, and ACE indices, along with rarefaction curves, were calculated and visualized using the MicrobiomeAnalystR platform, implemented through the phyloseq, ggplot2, and microbiomeseq R packages [15,16]. Microbial community structure was assessed using β-diversity analyses, employing Principal Coordinate Analysis (PCoA) based on Bray–Curtis dissimilarity and weighted UniFrac distances derived from pairwise sample comparisons. Statistical significance of between-group differences in community structure was evaluated using permutational multivariate analysis of variance (PERMANOVA).

Microbial association networks at the species level were inferred using the SparCC algorithm [17], which is specifically designed to account for compositionality in microbiome data through log-ratio transformations and iterative estimation of correlation structure. In parallel, Sparse Inverse Covariance Estimation for Ecological Association and Statistical Inference (SPIEC-EASI) was applied to infer global microbial interaction networks using graphical models [18]. Only robust associations were retained, defined as correlations with an absolute correlation coefficient (|r| > 0.6) and statistical significance (*p* < 0.05). Spearman’s rank correlation was used to define valid co-occurrence events, and SparCC-derived pseudo-*p*-values were calculated within the MicrobiomeAnalystR framework. Differential abundance analysis was performed using the DESeq2 package [19] and Microbiome Multivariable Association with Linear Models [MaAsLin2] [20] implemented in R (version 4.2) to identify bacterial taxa exhibiting significant changes at the species level. For inclusion in the analysis, taxa were required to have a relative abundance greater than 0.1% and to be present in more than 50% of samples in at least one group, unless otherwise specified.

### 2.5. Statistical Analysis

Group-wise comparisons of continuous variables were conducted using two-tailed Mann–Whitney U tests, while Kruskal–Wallis tests were applied for comparisons involving more than two categories. Differential abundance was quantified using log_2_ fold change values, and correction for multiple testing was performed using the Benjamini–Hochberg procedure (Q = 0.1, corresponding to FDR < 10%) [21]. Taxa were considered significantly differentially abundant if the adjusted *p*-value was < 0.05.

### 2.6. Ethical Statement

The study had been extensively reviewed and approved by the Ethical Committee of the Faculty of Dentistry, Universitas Trisakti, with the approval number 36/EthicalApproval/FKGUI/VII/2024.

## 3. Results

### 3.1. Vitamin D Levels Remain Stable Across Stunting Status and Intervention Groups

**Figure 1:**
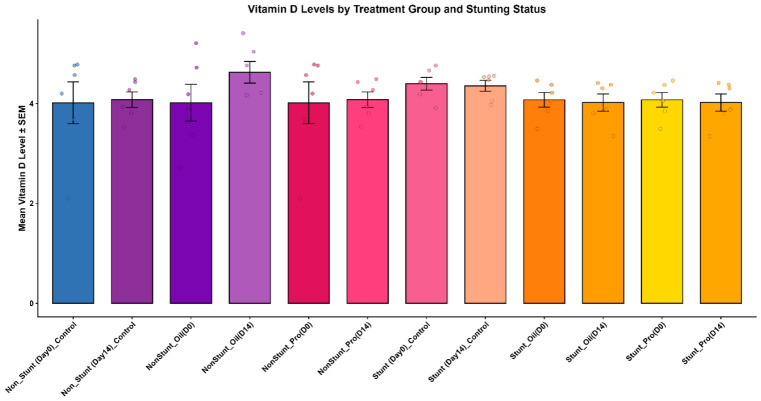
Distribution of vitamin D levels across stunting status and intervention groups. Boxplots show serum vitamin D concentrations at baseline (Day 0) and Day 14, stratified by stunting status (non-stunted and stunted) and intervention group (Control, Essential Oil, Probiotic). Each box represents the median and interquartile range (IQR), with whiskers indicating the minimum and maximum values; individual data points are overlaid. No significant differences were observed across stunting status, intervention, or time (overall ANOVA *p* = 0.224). Vitamin D levels remained comparable between groups and stable over the 14-day study period.

Vitamin D levels did not differ significantly by stunting status, treatment group, or time point (Figure 1). The average values were similar across all groups, ranging from about 4.01 to 4.62 units. Statistical analysis showed no significant effects of stunting status (*p* = 0.892), interaction group (*p* = 0.596), or time (*p* = 0.491). Although small changes were observed between Day 0 and Day 14 in some groups, these differences were minor and not statistically significant. Overall, vitamin D levels remained stable over the 14-day study period, regardless of stunting status or intervention.

### 3.2. Comparisons of Papilla Bleeding Index (PBI) and Oral Hygiene Index Score (OHI-S) Between Stunting and Non-Stunting Subjects Before and After 14-Day Intervention

**Figure 2:**
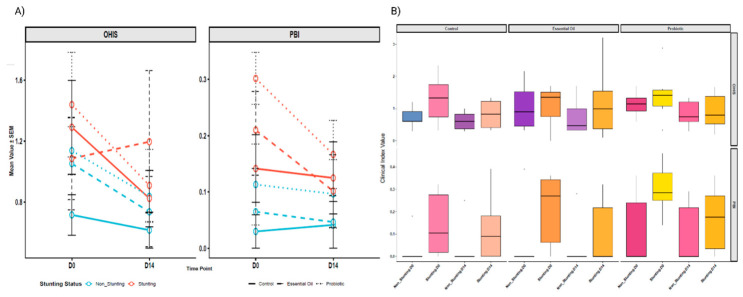
Clinical indices according to stunting status and intervention over 14 days. (**A**) Longitudinal changes in Oral Hygiene Index Simplified (OHIS) and Papillary Bleeding Index (PBI) at baseline (Day 0) and Day 14, stratified by stunting status (non-stunted, blue; stunted, red) and treatment (Control, Essential Oil, Probiotic). Data are shown as mean ± SEM. OHIS demonstrated modest reductions over time without significant main or interaction effects. In contrast, PBI showed a significant main effect of stunting status (*F* = 10.57, *p* = 0.002). (**B**) Distribution of OHIS and PBI across all group combinations. Boxplots indicate median and interquartile range. Higher PBI values were consistently observed in stunted participants at baseline.

Longitudinal assessment of clinical indices demonstrated baseline differences according to stunting status, with comparatively modest effects attributable to the 14-day interventions. As shown in Figure 2A, OHI-S values exhibited a downward trend from Day 0 to Day 14 across most groups, particularly within the probiotic group, these reductions were not statistically significant. There was no significant effect of stunting status (*F* = 3.30, *p* = 0.074), intervention type (*F* = 0.74, *p* = 0.481), or time (*F* = 3.12, *p* = 0.083). Additionally, there were no significant interaction effects between these factors.

In contrast, PBI showed a significant effect of stunting status (*F* = 10.57, *p* = 0.002), indicating that children with stunting had higher gingival inflammation compared with non-stunted children, regardless of intervention or time point. The effect size was moderate (generalized eta squared = 0.15). Correlation analysis confirmed this relationship (r = 0.36, *p* = 0.0017), while no significant correlations were observed for interventions or time.

The combined distribution plot (Figure 2B) further illustrates that the greatest inflammatory burden was observed in the Stunt-Probiotic group (D0) subgroup, with mean PBI values approximately threefold higher than those of the Non-Stunt (Day0) Control group. Although several pairwise comparisons involving the Stunt-Probiotic group (D0) reached nominal significance, these differences did not remain significant following multiple-comparison adjustment.

Collectively, these findings indicate that baseline inflammatory disparities are primarily driven by stunting status rather than short-term intervention effects. While probiotic and essential oil interventions were associated with numerical reductions in both OHI-S and PBI over 14 days, particularly among stunted children, these trends did not translate into statistically robust interventions-by-time interactions within the study period.

### 3.3. Taxonomic Composition and Microbial Interaction Networks

#### 3.3.1. Genus-Level Taxonomic Composition Across Stunting Status, Dietary Intervention, and Time

**Figure 3:**
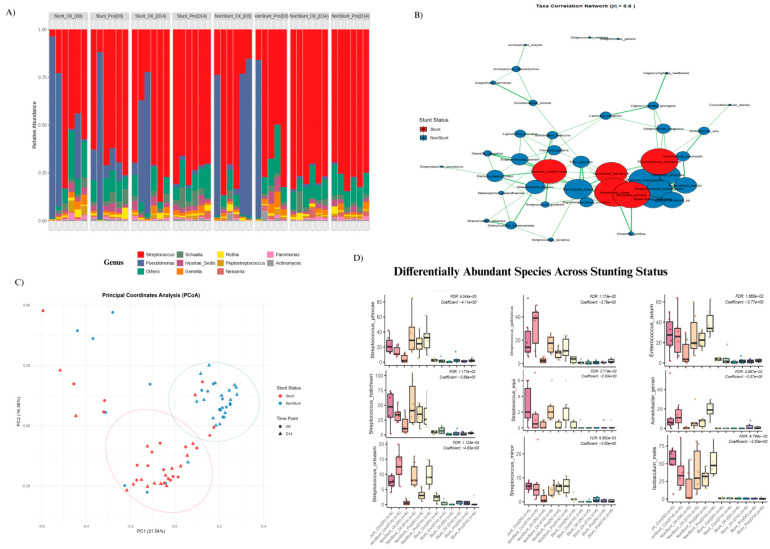
Genus-level taxonomic composition and SparCC-based microbial interaction networks across stunting status. (**A**) Stacked bar plots showing relative abundances of dominant bacterial genera across stunted and non-stunted groups at baseline (D0) and post-intervention (D14) under oil and probiotic supplementation. Genera with relative abundance < 0.5% were grouped as “Others.” (**B**) Taxa correlation network constructed using SparCC correlations (|r| > 0.6, *p* < 0.05). Node size represents degree centrality, and node color indicates association with stunted (red) or non-stunted (blue) status. Edges denote significant positive correlations. Highly connected taxa, including *Atopobium rimae*, *Fusobacterium nucleatum*, and *Bulleidia extructa*, highlight stunting-associated ecological hubs within the microbial community. (**C**) Microbial Community Structure, Stunting Status, and Intervention Groups. Principal Coordinates Analysis (PCoA) based on Bray–Curtis dissimilarity revealed distinct clustering of oral microbial communities according to both stunting status and intervention type. (**D**) Differential Abundance Across Stunting Status. MaAsLin2 and DESeq2 analyses revealed significant variation in the top nine differentially abundant species in comparison with the non-stunted group, as indicated (|log_2_ fold change| > 1, adjusted *p* < 0.05).

The stacked bar plot (Figure 3A) illustrated genus-level relative abundances across stunted and non-stunted groups at baseline (D0) and post-intervention (D14) under essential oil and probiotic supplementation. Across all samples, the microbial community was strongly dominated by Streptococcus, which accounted for 62.71% of the total relative abundance, establishing it as the core genus irrespective of stunting status or intervention. Pseudomonas represented the second most abundant genus (17.28%), followed by substantially lower contributions from Schaalia (2.66%), unclassified taxa (Incertae sedis, 2.25%), Gemella (1.98%), Rothia (0.97%), Peptostreptococcus (0.92%), Neisseria (0.71%), Parvimonas (0.66%), and Actinomyces (0.63%).

Despite this shared core oral structure, profiles of relative abundance presented significant inter-individual variability. This pattern was especially evident in the stunting groups, where non-*Streptococcus* genera contributed a higher and more heterogeneous proportion of the community. Notably, samples from the stunting group displayed increased representation of anaerobic and pathobiont-associated genera, such as *Parvimonas*, *Peptostreptococcus*, and *Actinomyces*. On the other hand, non-stunting groups showed a more stable dominance of *Streptococcus*, with reduced dispersion across samples. Temporal comparisons (D0 vs. D14) revealed no uniform taxonomic shifts attributable solely to the interventions of essential oil or probiotic. This suggests that the short-term interventions did not override or alter the baseline community structure. Overall, these findings indicate that while a *Streptococcus*-dominated microbiome is conserved across conditions, stunting status is linked with increased compositional instability and enrichment of low-abundance anaerobic taxa.

#### 3.3.2. SparCC-Based Taxa Correlation Network Reveals Stunting-Associated Keystone Taxa

To investigate microbial interaction structure beyond relative abundance, SparCC correlation analysis was performed using a stringent threshold (|r| > 0.6, *p* < 0.05), generating a taxa correlation network highlighting putative ecological relationships. Node size reflects degree centrality, while node color indicates stunting status association (Figure 3B).

The correlation network identified multiple highly connected taxa functioning as putative ecological hubs within the oral microbial community. *Atopobium rimae* exhibited the highest degree of centrality (*degree* = 14), followed by *Fusobacterium nucleatum* (*degree* = 13) and *Bulleidia extructa* (*degree* = 13), underscoring their central roles in shaping microbial co-occurrence patterns. Additional highly connected nodes included *Gemella morbillorum* (*degree* = 12), an uncultured bacterium (*degree* = 11), *Ihubacter massiliensis* (*degree* = 11), and *Parvimonas micra* (*degree* = 10). Notably, several of these taxa—particularly *F. nucleatum*, *P. micra*, *Tannerella forsythia* (*degree* = 9), and *Porphyromonas endodontalis* (*degree* = 9)—are well-established oral pathobionts implicated in inflammation and periodontal disease.

Importantly, high-degree nodes were predominantly associated with the stunted group and formed densely interconnected clusters, suggestive of cooperative or syntrophic interactions among anaerobic taxa. In contrast, taxa enriched in non-stunted samples displayed fewer connections and occupied more peripheral positions within the network, consistent with a less interconnected microbial community architecture. The inferred association network comprised 41 nodes and 112 edges, yielding a network density of 0.1366 and an average node degree of 5.46, indicative of a moderately connected system. The observed modularity (0.4176) further supports the presence of distinct community modules, with stunting-associated taxa contributing disproportionately to tightly linked subnetworks. Collectively, these findings demonstrate that stunting is associated not only with shifts in taxonomic composition but also with a pronounced reorganization of microbial interaction networks toward a more interconnected, and potentially dysbiotic, ecological state.

While genus-level abundance profiles (Figure 3A) reveal dominance by *Streptococcus* across all groups, network-based analysis (Figure 3B) uncovers substantial differences in microbial interaction topology linked to stunting. The enrichment of highly connected anaerobic and pathogenic taxa in stunted individuals suggests that microbial dysbiosis in stunting may be driven less by changes in dominant taxa and more by restructuring of low-abundance but highly interactive community members.

#### 3.3.3. Global β-Diversity Structure Across Stunting Status

Principal Coordinates Analysis (PCoA) based on Bray–Curtis dissimilarity revealed distinct clustering of oral microbial communities according to both stunting status and intervention type (Figure 3C). The first two principal coordinates accounted for [21.52] % (PC1) and [16.36] % (PC2) of total community variance, respectively. Along PC1, the primary axis of separation stunted, and non-stunted individuals formed clearly segregated clusters, indicating that host nutritional status represents the dominant driver of oral microbial community structure. Within each stunting category, secondary separation along PC2 distinguished probiotic-treated from oil-treated groups, demonstrating measurable intervention-specific restructuring of the oral ecosystem. Ellipse contours (95% confidence intervals) showed tight intra-group clustering with minimal overlap between experimental conditions, confirming statistically significant differences in overall community composition (PERMANOVA: R^2^ = [0.0987], *p* < 0.001).

Differential abundance analysis using complementary frameworks (MaAsLin2 and DESeq2) identified a distinct set of oral microbial taxa contributing to the observed community-level differences between stunted and non-stunted individuals (Figure 3D). Applying stringent significance thresholds (|log_2_ fold change| > 1, adjusted *p* < 0.05), stunted individuals exhibited a pronounced depletion of *Streptococcus* species relative to non-stunted controls. Among the most significantly differentially abundant taxa, *Isobaculum melis* showed the strongest reduction in stunted samples (log_2_FC = −4.28, adjusted *p* = 9.68 × 10^−3^), followed by *Enterococcus avium* (log_2_FC = −2.82, adjusted *p* = 9.68 × 10^−3^). Notably, multiple *Streptococcus* species were significantly depleted, including *Streptococcus phocae* (log_2_FC = −2.92, adjusted *p* = 9.68 × 10^−3^) and *Streptococcus halichoeri* (log_2_FC = −2.89, adjusted *p* = 9.68 × 10^−3^), highlighting a consistent reduction in this genus species in stunted individuals. Collectively, these findings indicate that stunting is associated with selective depletion of commensal taxa, particularly *Streptococcus* spp., which may contribute to the altered microbial ecology observed in this population.

#### 3.3.4. Global β-Diversity Structure Across Stunting Status Associated with Dietary Intervention

**Figure 4:**
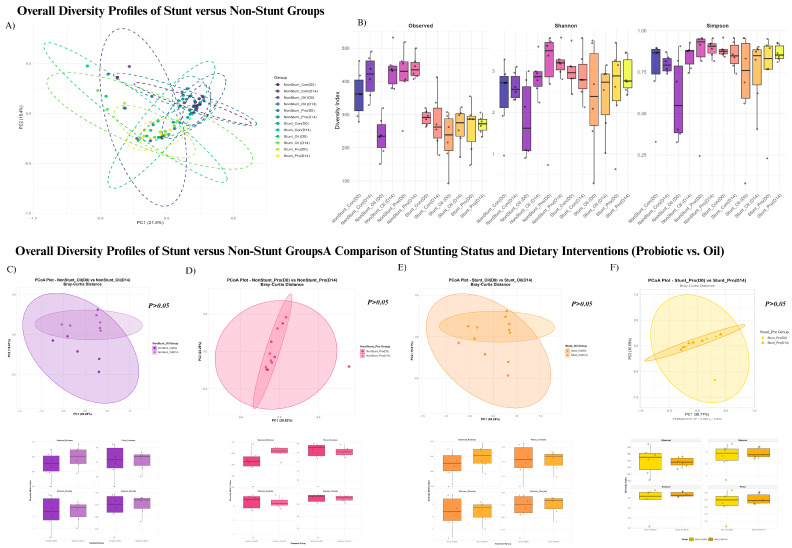
Overall diversity profiles of stunted versus non-stunted groups following dietary interventions. (**A**) PCoA based on Bray–Curtis dissimilarity showing global microbial community structure across stunted and non-stunted groups, dietary interventions (probiotic vs. oil), and time points (D0 and D14). Ellipses represent 95% confidence intervals. (**B**) Alpha diversity indices (Observed richness, Shannon, and Simpson) comparing groups across stunting status, intervention type, and time points. Boxplots show median and interquartile range, with individual data points overlaid. (**C**–**F**) PCoA plots comparing baseline (D0) and post-intervention (D14) samples within each group: (**C**) non-stunted oil, (**D**) non-stunted probiotic, (**E**) stunted oil, and (**F**) stunted probiotic. Corresponding alpha diversity comparisons are shown below each PCoA. Statistical significance for β-diversity was assessed using PERMANOVA; all comparisons were non-significant (*p* > 0.05).

Principal coordinate analysis (PCoA) based on Bray–Curtis dissimilarity revealed substantial overlap in overall microbial community structure across stunted and non-stunted groups, irrespective of dietary intervention (probiotic vs. oil) and sampling time points (D0 and D14) (Figure 4A). Samples clustered broadly along the first two principal coordinates, which explained approximately 21.6% (PC1) and 16.4% (PC2) of the total variance, respectively. Although distinct ellipses were observed for some group time combinations, no clear separation was evident between stunted and non-stunted cohorts, suggesting that neither stunting status nor short-term dietary intervention induced large-scale restructuring of the gut microbial community. The dispersion patterns indicate considerable inter-individual variability, consistent with a heterogeneous microbiome background within each group.

#### 3.3.5. Alpha-Diversity Comparisons Across Stunting Status Associated with Dietary Intervention

Alpha diversity was assessed using observed richness, Shannon diversity, and Simpson diversity indices (Figure 4B). Non-stunted control and probiotic groups generally exhibited higher observed richness compared with stunted groups at baseline and after intervention. Shannon diversity followed a similar trend, with non-stunted individuals displaying greater community evenness, whereas stunted groups showed reduced diversity and higher variability. Simpson diversity values were broadly high across all groups, indicating dominance by a limited number of taxa. However, stunted groups, particularly at baseline, exhibited lower Simpson indices, reflecting reduced evenness. Collectively, these results suggest that stunting is associated with lower microbial richness and diversity, while short-term probiotic or oil supplementation did not substantially restore alpha diversity within the intervention window.

#### 3.3.6. Non-Stunted Group—Essential Oil Intervention (D0 vs. D14)

PCoA analysis of non-stunted individuals receiving oil supplementation showed strong overlap between baseline (D0) and post-intervention (D14) samples, with no significant separation (PERMANOVA, *p* > 0.05) (Figure 4C). This indicates that oil supplementation did not significantly alter overall microbial community composition in non-stunted individuals over the 14-day period. Consistent with the β-diversity findings, alpha diversity indices (observed richness, Shannon, and Simpson) remained stable between time points, suggesting resilience of the microbiome in non-stunted individuals to this dietary intervention.

#### 3.3.7. Non-Stunted Group—Probiotic Intervention (D0 vs. D14)

Similarly, probiotic supplementation in non-stunted individuals did not result in significant shifts in community structure, as evidenced by overlapping PCoA clusters and non-significant PERMANOVA results (*p* > 0.05) (Figure 4D). Alpha diversity metrics remained comparable between baseline and post-intervention samples, indicating that the probiotic did not disrupt or substantially enhance microbial diversity in an already stable non-stunted microbiome. These findings suggest that probiotic effects in non-stunted individuals may be subtle or functionally targeted rather than reflected in gross compositional changes.

#### 3.3.8. Stunted Group—Essential Oil Intervention (D0 vs. D14)

In stunted individuals receiving oil supplementation, PCoA plots demonstrated overlapping but more dispersed clusters relative to non-stunted groups, indicating higher inter-individual variability. No statistically significant separation between D0 and D14 samples was observed (*p* > 0.05) (Figure 4E), suggesting that oil supplementation alone was insufficient to induce detectable changes in overall community structure. Alpha diversity indices showed modest, non-significant increases post-intervention, implying limited recovery of microbial diversity within the short intervention period.

#### 3.3.9. Stunted Group—Probiotic Intervention (D0 vs. D14)

PCoA analysis of the stunted probiotic group also revealed no significant compositional shift between baseline and post-intervention samples (*p* > 0.05), despite a slight directional clustering along PC1 (Figure 4F). Alpha diversity indices exhibited marginal improvements in observed richness and Shannon diversity at D14, although these changes did not reach statistical significance. This suggests that while probiotic supplementation may exert modest modulatory effects in stunted individuals, short-term intervention does not fully overcome the underlying microbiome dysbiosis associated with stunting.

Across all analyses, stunting status was associated with reduced microbial richness and diversity, whereas short-term dietary interventions, either probiotics or essential oils, did not produce significant shifts in global microbial community structure. The absence of strong β-diversity separation suggests microbiome resilience and highlights that functional or taxon-specific changes, rather than large-scale compositional restructuring, may underlie any intervention effects.

## 4. Discussion

There are many factors to determine how stunting children can have different oral health profiles, along with the treatment given. Specifically in this study, we examine vitamin D levels, along with oral health indices, and the oral microbiome.

Vitamin D emerged as a relevant immunomodulatory factor within this ecological framework. Through activation of Vitamin D binding receptor (VDR), Vitamin D regulates expression of antimicrobial peptides, maintains mucosal barrier integrity, and modulates inflammatory responses, all of which influence microbial community composition [22,23]. Changes in salivary vitamin D levels following either probiotic or essential oil mouthwash intervention were not statistically significant in this pilot study (Figure 1). Meanwhile, the non-stunting group tended to have higher means compared to the stunting counterpart. This pattern aligns with the known association between stunting and compromised vitamin D status, which reduces host receptivity to beneficial microbial colonization [22]. Existing evidence suggests that adequate vitamin D status is associated with increased microbial diversity, expansion of beneficial commensal taxa, and suppression of inflammation-associated bacteria [22,24]. Therefore, with the supplementation of probiotics and/or essential oils, the presence of adequate vitamin D may enhance host receptivity to microbial recolonization, optimize immune–microbiome crosstalk, and facilitate a stable return to eubiosis. Even in the absence of a large post-intervention shift in salivary vitamin D, maintenance of adequate status likely contributes to the observed improvements in non-stunting children. The interplay between nutritional status, vitamin D-mediated immune regulation, and microbial network dynamics warrants further investigation in larger, longitudinal studies.

This current study attempts to convey that childhood stunting significantly affects the oral health indices, especially the papillary bleeding index. Data showed that stunting has a significant detrimental impact on gingival health since stunted children consistently had higher Papillary Bleeding Index (PBI) scores than their counterparts in each intervention arm (Control, Essential Oil, and Probiotic). According to the significant main effect of stunting status (*F* = 10.57, *p* = 0.002), increased gingival inflammation and bleeding are independently produced by chronic malnutrition. This is certainly linked to the impaired systemic immunity, reduced salivary protective capacity, and higher susceptibility to plaque-induced pathology [25]. All this is supported by Panel B boxplots showing that in stunted subjects, there is at least a larger baseline PBI median and interquartile range independent of the kind of therapy (Figure 2). On the contrary, the OHIS value of all groups decreased slightly during the 14 days, without significant main effects or stunting-by-intervention interactions. This indicates that the short-term combined treatments did not target the primary nutritional deficiencies in oral tissues, and the increase in plaque control was only subtle. In total, the findings indicate that stunting is a significant risk factor for lower periodontal clinical indices. However, these findings also underscore the need for sustained, integrated nutritional–oral health interventions extending well beyond 14 days to meaningfully address this reciprocal relationship in at-risk pediatric populations, and future studies with longer intervention periods and larger sample sizes are warranted to confirm these observations. It is important to acknowledge that the study population (aged 8–12 years) is in a phase of mixed dentition and pre-pubertal hormonal transition. Both of these biological factors are independently known to transiently increase gingival bleeding susceptibility due to altered immune responses and hormonal influences on periodontal vascularity. Therefore, the elevated PBI values observed in stunted children should be interpreted with caution, as the contribution of mixed dentition and pre-pubertal hormonal fluctuations to gingival inflammatory responses cannot be excluded from the present cross-sectional comparisons. Future studies should stratify participants by dentition stage and hormonal maturation status, or employ adjusted models, to more precisely isolate the contribution of nutritional stunting to periodontal outcomes.

The present study provides evidence that childhood stunting is associated with fundamental alterations in oral microbiome stability and microbial interaction architecture rather than overt shifts in dominant bacterial taxa alone. While the oral microbiome across all participants remained largely *Streptococcus*-dominant, consistent with its established role as a primary colonizer [26], stunted children exhibited greater inter-individual variability, reduced microbial diversity, and enrichment of low-abundant, strict anaerobic, and pathobiont-related taxa. Notably, genera such as *Parvimonas*, *Peptostreptococcus*, and Actinomyces were enriched in stunted children (Figure 3). These findings suggest that stunting is linked to a destabilized oral ecosystem characterized by increased ecological heterogeneity and reduced resilience features that are hallmarks of microbial dysbiosis. Rather than representing a complete taxonomic replacement, these changes indicate a shift in ecological balance, whereby reduced dominance of health-associated commensal permits expansion of opportunistic taxa within the ecological niche. Such restructuring aligns with contemporary ecological models of oral dysbiosis, which emphasize instability and altered microbial interactions over the mere presence or absence of specific pathogens [27,28].

Network-based SparCC analysis further strengthened this interpretation by revealing the pronounced reorganization of microbial interaction architecture seen in stunted children. Keystone taxa identified as being highly connected to each other include *Fusobacterium nucleatum*, *Parvimonas micra*, *Tannerella forsythia*, and *Porphyromonas endodontalis*, which are formed into dense interconnected hubs, with the stunted group being heavily associated with these hub types. These taxa are well recognized for their role in the pathogenicity of periodontal disease. They play an important role in the periodontal biofilm maturation process and inflammatory response through synergistic mechanisms such as co-aggregation and metabolic cross-feeding [29,30]. The high centrality and modular clustering of these keystone taxa foster a microbial network structure conducive to cooperative pathogenicity even without significant changes in the relative abundance of microbial species [31]. The findings of this study may support the emerging paradigm of new ecological models, which may indicate that disease risk is not only dependent on the microbial composition but also on the topology and dynamics of microbial interaction networks.

Beta-diversity analysis further indicated that host nutritional status was a major factor influencing oral microbial community structure. Clear separation between stunted and non-stunted individuals along primary principal coordinate axes suggests that chronic undernutrition exerts a persistent ecological pressure on the oral microbiome. Mechanistically, malnutrition has been associated with impaired salivary secretion, altered salivary composition, compromised mucosal immunity, and deficiency of micronutrients, all of which may impair host control of microbial colonization and biofilm regulation [32,33]. One interesting finding is the significant decrease in numerous *Streptococcus* species from the mouths of stunted individuals. These bacteria are essential in buffering the mouth’s pH, competing against other microorganisms for space, and educating the immune system. Loss of these commensal species may reduce ecological resistance to pathobiont expansion, contributing to the observed network instability and dysbiotic state [34].

In contrast, short-term application of probiotics or short-term application of essential oils did not result in global reconfiguration of the oral microbiome as evidenced by largely overlapping PCoA plots and the statistically insignificant PERMANOVA results across all intervention groups (Figure 4). Alpha diversity metrics similarly remained largely unchanged, with only modest, non-significant increases observed in a subset of stunted individuals who received probiotics [35,36]. These findings are consistent with previous studies demonstrating the inherent resilience of established oral microbial communities, particularly to the short-term interventions. It is likely that probiotic and phytochemical interventions exert more subtle or functionally targeted effects such as modulation of gene expression, metabolite production, and host–microbe signaling pathways, which are not readily captured by 16S taxonomic profiling [37].

The reduced richness and evenness in stunted children relative to control subjects further suggest that oral dysbiosis in this population reflects long-term ecological constraints imposed by chronic malnutrition rather than transient environmental perturbations. Restoration of microbial balance in such contexts requires sustained nutritional rehabilitation, immune maturation, and prolonged ecological pressure to re-establish stable commensal networks.

The present findings indicate that short-term dietary interventions alone may not be sufficient to reverse this established ecological dysbiotic state associated with stunting, underscoring the need for integrated, long-term strategies.

Compared with prior studies [38,39,40], which have primarily examined probiotic or essential oil interventions in isolation (often reporting as transient reductions in pathogen load or inflammation), this study contributes a broader ecological perspective. While earlier works noted temporary microbial suppression or modest clinical benefits, they rarely documented sustained shifts in microbial community structure or diversity following long-term intervention. Rather than reporting in contrast, the synergy model predicts progressive microbiome restructuring supported by host–microbial interactions, immune modulation by vitamin D, and ecological stability. Rather than demonstrating immediate therapeutic effects, the findings highlight the dominant role of host ecology in shaping oral microbiome stability and suggest that meaningful microbiome modulation in stunting children may depend on sustained, multi-level interventions.

Collectively, the findings of the study contribute to the growing evidence regarding the oral-systemic interaction in nutritionally impaired children. Especially in Indonesia and other low- to middle-income countries, stunting represents an indicator of chronic undernutrition and socioeconomic disadvantage groups. Hence, stunting has been associated with increased risk of dental caries, altered salivary composition, and shifts in the oral microbiome [1,2,3]. In broader populations, microbial dysbiosis has been associated with low-grade systemic inflammation and altered energy metabolism [41]. By demonstrating that stunting is associated with reconfigured microbial interaction networks and reduced ecological stability, this study supports the concept that early nutritional adversity may imprint lasting effects on host–microbiome interactions. Notably, while the short-term probiotic and essential oil mouthwash interventions did not produce statistically significant shifts in microbiome composition, the directional trends observed suggest a potential for microbiome-targeted adjunctive strategies to complement nutritional rehabilitation in high-risk groups.

However, whether microbiome restoration in stunted children could attenuate long-term metabolic risk requires dedicated longitudinal studies with appropriate systemic endpoints, including anthropometric and metabolic biomarker monitoring, which were beyond the scope of the present pilot investigation. Future studies integrating functional metagenomics and host immune profiling will be critical to elucidate the mechanistic pathways linking undernutrition, microbial network topology, and long-term oral and systemic health outcomes.

The present pilot study has several limitations that should be considered when interpreting its findings and that inform the direction of future research. First, although 72 participants were enrolled (36 stunted, 36 non-stunted), allocation across three parallel intervention arms resulted in modest subgroup sizes, reducing statistical power to detect intervention effects within a dataset inherently characterized by high inter-individual microbiome variability. Second, the 14-day intervention period is likely insufficient to induce meaningful compositional shifts in a resilient, established oral microbial ecosystem, particularly in children with chronic and long-standing nutritional deficits. Third, the open-label design, conducted without blinding of participants or examiners, introduces the possibility of performance and detection bias, particularly for subjective clinical indices such as OHI-S and PBI. Fourth, recruitment from a single geographic site in Indonesia limits the generalizability of findings to other age groups, ethnic backgrounds, and socioeconomic contexts. Fifth, reliance on 16S rRNA amplicon sequencing provides taxonomic resolution but does not capture functional microbial activity, gene expression profiles, or the mechanistic dimensions of host–microbiome interactions. Sixth, the open-label approach was selected to ensure transparency with the parents/guardians regarding the intervention consumed by their children, which was ethically important in this pediatric study setting, yet may introduce potential result bias due to the lack of randomization and blinding.

Future studies should address these limitations through adequately powered, multi-center randomized controlled trials incorporating double-blinding, longer intervention periods of at least three to six months, and add more diverse pediatric populations, whilst maintaining appropriate ethical disclosure to the parents/guardians. A collection of dietary habits, socioeconomic background, and developmental status can also be added for more encompassing results. Integration of multi-omics approaches, including shotgun metagenomics, metatranscriptomics, and metabolomics, would substantially advance mechanistic understanding of how nutritional status shapes oral microbial ecology and how targeted interventions may restore eubiosis. Such studies would provide the rigorous evidence base needed to develop effective, integrated nutritional and oral health strategies for children affected by stunting.

## 5. Conclusions

Within the limitations of the study, we concluded that childhood stunting was associated with reduced oral microbiome diversity, enrichment of pathobiont-associated taxa, and reorganized microbial interaction networks that favor dysbiosis. Salivary vitamin D levels remained stable and did not differ significantly across groups or time points in this pilot study. Short-term 14-day interventions with probiotics or essential oil mouthwash produced modest, non-significant improvements in clinical indices (OHI-S, PBI) and no significant global shifts in microbial community structure, consistent with the inherent resilience of established oral microbiomes. These findings underscore that restoring oral eubiosis in stunted children likely requires sustained, multi-component nutritional and microbiome-targeted strategies. Future longitudinal studies incorporating systemic metabolic profiling and functional metagenomics are needed to elucidate the mechanistic links between undernutrition, microbial network topology, and long-term oral and systemic health outcomes.

## Data Availability

The data presented in this study are available on request from the corresponding author. The raw FASTQ sequences are publicly available and have been deposited in the NCBI Sequence Read Archive (SRA) under accession number PRJNA1463604.
